# COX-2 gene rs689466 polymorphism is associated with increased risk of colorectal cancer among Caucasians: a meta-analysis

**DOI:** 10.1186/s12957-020-01957-x

**Published:** 2020-07-30

**Authors:** Yong-Chen Zhang, Hui Zhao, Chen Chen, Mohammad Amzad Ali

**Affiliations:** 1 Department of Laboratory Medicine, The Second Hospital of Nanjing, Nanjing University of Chinese Medicine, Nanjing, 210003 China; 2 Department of General Surgery, Affiliated Hospital of Jiangnan University, Wuxi, China; 3 Department of Thoracic Surgery, The Affiliated Huaian No.1 People’s Hospital of Nanjing Medical University, Huaian, Jiangsu China; 4 Department of Casualty (emergency), Pandit Madan Mohan Malviya government hospital Malviya Nagar, New Delhi, India

**Keywords:** COX-2, Colorectal cancer, Susceptibility, Polymorphism

## Abstract

**Background:**

Several studies have reported the Cyclooxygenase 2 (COX-2) rs689466 polymorphism as a susceptibility locus of colorectal cancer (CRC), but their findings are inconsistent. Thus, this meta-analysis was performed to more accurately identify the effects of this polymorphism on CRC risk.

**Methods:**

Potential case-control studies on EMBASE, Google Scholar, Web of Science, Cochrane Library, and PubMed were searched. The strength of association was quantified by pooled odds ratio and 95% confidence interval. Totally 16 articles involving 8998 cases and 11,917 controls were included.

**Results:**

None of the five tested genetic models revealed an association between rs689466 polymorphism and CRC risk. Stratified analysis by ethnicity uncovered a positive association between this polymorphism and higher CRC risk in Caucasians, but not in Asians. In addition, we found that high expression of COX-2 was associated with better overall survival for all CRC patients.

**Conclusion:**

To sum up, the COX-2 rs689466 polymorphism may be related with susceptibility to CRC in Caucasians. This finding should be verified by larger-size studies with different ethnic groups.

## Introduction

Colorectal cancer (CRC), the second largest cause of cancer-induced death in the world [1], was estimated to cause 135,430 new cases and 50,260 deaths in the USA in 2017 [2]. However, the cause of CRC is unknown yet. Risk of CRC is significantly associated with diet, cigarette smoking, drinking, and other factors [3, 4]. The development of CRC also involves genetic factors [5].

Cyclooxygenases (COXs), including two isoforms of COX-1 and COX-2 identified so far, are critical in transforming arachidonic acid to the precursor of prostaglandins—prostaglandin H2 [6]. COX-2 overexpression in CRC tissues was associated with worse overall survival of CRC [7]. Paeonol, a COX-2 inhibitor, constrains prostaglandin E2 (PGE2) generation and COX-2 expression, thereby preventing human CRC cells from tumors [8]. COX-2 downregulation considerably eliminates the development, motion, and invasion of colon cancer [9]. These observations suggest COX-2 is pivotal in CRC development.

COX-2 containing 10 exon counts is located in the chromosome 1q31.1. Rs689466 polymorphism is in the promoter zone of COX-2 gene. The association between rs689466 polymorphism and CRC risk has been explored extensively [10–25]. However, the findings of these studies were inconclusive and inconsistent, which may be attributed to the small sample sizes, clinical heterogeneity, and ethnic differences. To solve the inconsistence among these studies, we designed this meta-analysis to clarify the potential association between COX-2 gene rs689466 polymorphism and CRC risk.

## Materials and methods

### Literature search and inclusion criteria

Two reviewers systematically and independently searched EMBASE, Google Scholar, Web of Science, Cochrane Library, and PubMed to find potential studies without any restriction. The key words included “polymorphism”, “single nucleotide polymorphism” or “SNP”, “colorectal cancer”, “colorectal tumor” or “CRC”, and “Cyclooxygenase-2” or “COX-2”. References of identified studies were manually screened to search any omitted article.

The inclusion criteria were (1) case-control studies, (2) enough data for computation of pooled odds ratio (OR) with 95% confidence interval (CI), (3) evaluation of association between COX-2 rs689466 polymorphism and CRC risk, and (4) target at humans.

### Prognosis analysis

OncoLnc (http://www.oncolnc.org/) website was used to evaluate prognostic value of the mRNA expression (high vs. low expression, separated by 50%) of COX-2 gene. We analyzed the overall survival (OS) of CRC patients by calculating Log rank *p* value and hazard ratio (HR) with 95% confidence intervals.

### Expression analysis

We also performed the expression quantitative trait loci (eQTL) analysis using GTEx portal web site (http://www.gtexportal.org/home/) to predict potential associations between the SNPs and gene expression levels.

### Data isolation and quality assessment

Based on the inclusion criteria, two reviewers independently extracted the data of interest, including ethnicity, sample sizes (cases, controls), cancer type, name of first author, publication year, and country of origin. If data were unavailable in an article, we contacted the authors for relevant data. If more than one ethnicity were involved in one article, we collected genotype data separately.

The quality of each included study was assessed using the Newcastle-Ottawa Scale (NOS) [26]. Generally, a score from 5 to 9 stars indicates high methodological quality while a score from 0 to 4 means slow quality. Disagreements between the two reviewers were solved by discussion or consultancy with a third reviewer.

### Statistical analysis

Statistical analyses were carried out using Stata 11.0 (StataCorp, College Station, USA). Stratified analyses of ethnicity, source of control (SOC), Hardy–Weinberg equilibrium (HWE), and genotyping methods were also conducted. Regarding potential heterogeneity among studies, we defined significant heterogeneity at the levels *p* < 0.10 and *I*^2^ > 50%. A random-effect model was used in case of significant heterogeneity; otherwise, a fixed-effect model was used [27]. The effect on heterogeneity test and the stability of results were evaluated via sensitivity analysis by eliminating one study each time. HWE in the controls was examined via Pearson’s *χ*^2^ test. The positive findings were evaluated by calculating false-positive report probability (FPRP). An FPRP threshold of 0.2 and a prior probability of 0.1 were set to detect an OR for a correlation with the tested genotype. FPRP < 0.2 implied a possible relationship. Publication bias was tested by visually inspecting the symmetry of Begg’s funnel plot and assessing Egger’s test [28]. The Power and Sample Size Program software was used to calculate power and sample size. The following parameters are used: *α*, the type I error probability for a two-sided test; P0, the probability of exposure in controls; *N* is the number of case patients; *m*, the ratio of control to experimental subjects; *Ψ*, odd ratio of exposure in cases relative to controls. Statistical significance was set at *p* < 0.05.

## Results

### Characteristics of included articles

**Fig. 1:**
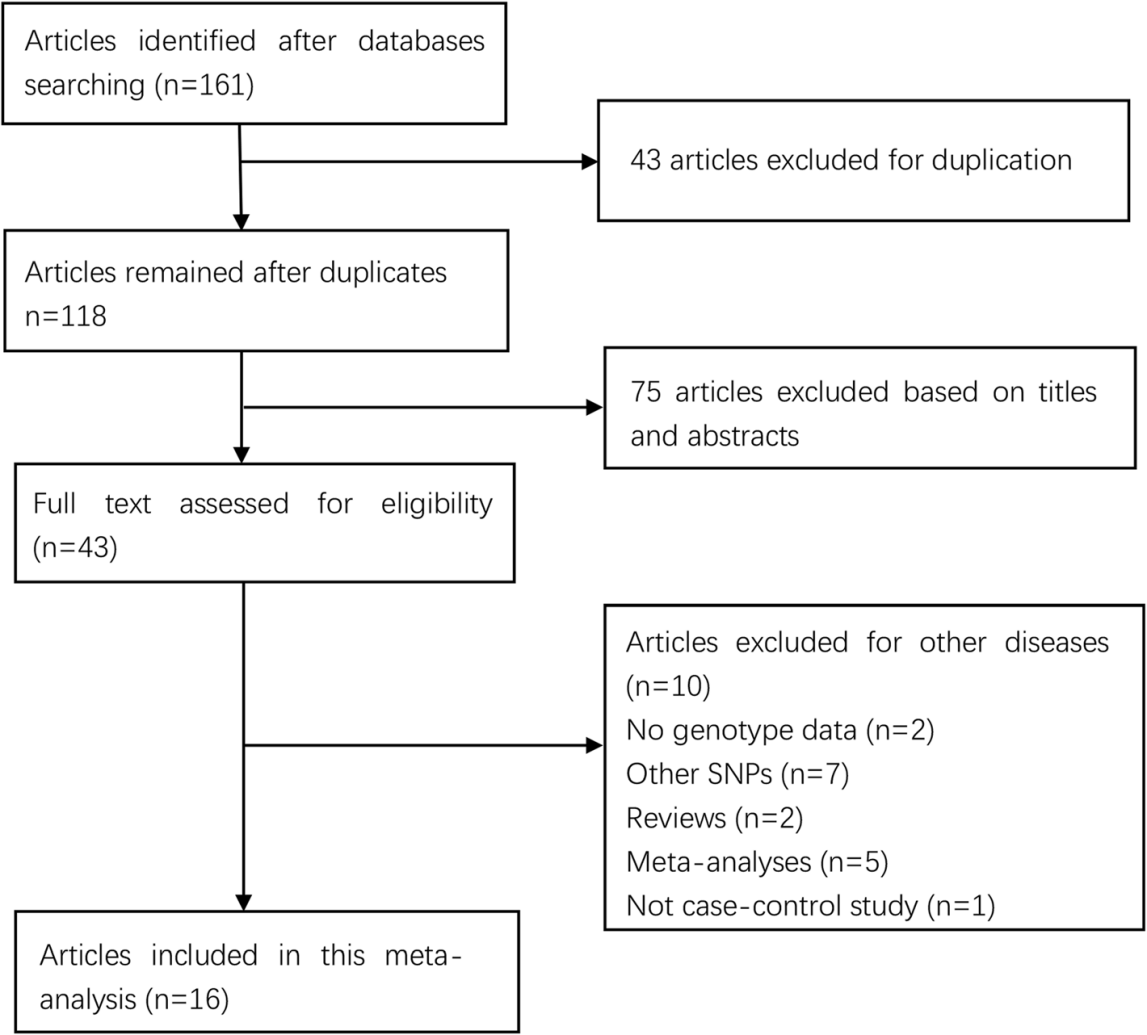
Selection for eligible papers included in this meta-analysis

The initial search returned 161 articles. Then, 43 duplicated articles were excluded, and 75 articles were omitted after title and abstract examination. Of the remaining 43 articles, full-text review denied 27 articles. Finally, 16 studies with 8998 cases and 11917 controls were included [10–25]. The process of article selection is illustrated in Fig. 1. The characteristics of the included studies are listed in Table 1. Two ethnicities were involved, including Caucasians (12 studies) [12–14, 16–21, 23–25] and Asians (4 studies) [10, 11, 15, 22]. Two studies failed to conform to HWE [15, 22]. The NOS scores range from 5 to 7 stars, suggesting that the included studies are all of high quality.

**Table 1:** Characteristic of studies on the association between COX-2 rs689466 polymorphism and colorectal cancer

Author and year	Country	Ethnicity	SOC	Genotyping method	Case			Control			***p*** values*	HWE	NOS
					AA	AG	GG	AA	AG	GG			
Siezen et al. [16], 2005	Netherlands	Caucasian	PB	Pyrosequencing	218	131	22	255	122	16	> 0.05	0.77	7
Siezen et al. [16, 17], 2006	Netherlands	Caucasian	PB	Pyrosequencing	410	191	29	665	354	61	> 0.05	0.13	6
Tan et al. [15], 2007	China	Asian	PB	PCR-RFLR	320	502	178	308	692	300	< 0.05	0.02	6
Andersen et al. [24], 2009	Denmark	Caucasian	PB	Taqman QPCR	230	116	13	482	258	25	0.88	0.177	7
Hoff et al. [23], 2009	Netherlands	Caucasian	PB	PCR-RFLR	213	101	12	232	124	13	0.99	0.471	8
Thompson et al. [14], 2009	USA	Caucasian	PB	Taqman	275	138	9	297	168	15	0.99	0.131	7
Pereira et al. [20], 2010	Portugal	Caucasian	HB	PCR-RFLR	70	43	4	177	73	6	0.078	0.634	7
Zhang et al. [10], 2012	China	Asian	HB	PCR-RFLR	77	216	50	62	184	94	< 0.05	0.09	6
Ruan et al. [11], 2013	China	Asian	HB	PCR-RFLR	34	67	29	39	53	28	0.847	0.232	6
Andersen et al. [25], 2013	Denmark	Caucasian	PB	PCR	587	313	47	1126	560	61	0.09	0.397	7
Li et al. [22], 2013	China	Asian	HB	PCR-RFLR	116	248	87	179	366	114	0.34	0.002	6
Makar et al. [21], 2013	USA	Caucasian	PB	Illumina	1529	742	90	2156	1005	130	> 0.05	0.343	8
Pereira et al. [19, 29], 2014	Portugal	Caucasian	PB	Taqman	143	85	15	323	133	16	0.076	0.614	7
Vogel et al. [12], 2014	Norway	Caucasian	PB	KASPTM	626	284	23	209	114	11	0.262	0.337	8
Shomaf et al. [18], 2015	Jordan	Caucasian	HB	PCR-RFLR	68	61	6	87	27	1	0.13	0.483	7
Tomitao et al. [13], 2017	Brazil	Caucasian	HB	Taqman	146	72	12	135	55	6	0.17	0.89	7

*HB* hospital-based controls, *PB* population-based controls, *SOC* source of controls, *PCR-RFLR* polymerase chain reaction-restriction fragment length polymorphism, *KASPTM* kompetitive allele-specific polymerase chain reaction, *QPCR* quantitative real-time polymerase chain reaction, *HWE* Hardy–Weinberg equilibrium, *NOS* Newcastle–Ottawa Scale

*p values for the association between COX-2 gene rs689466 polymorphism and colorectal cancer risk from original article

### Quantitative analysis

**Fig. 2:**
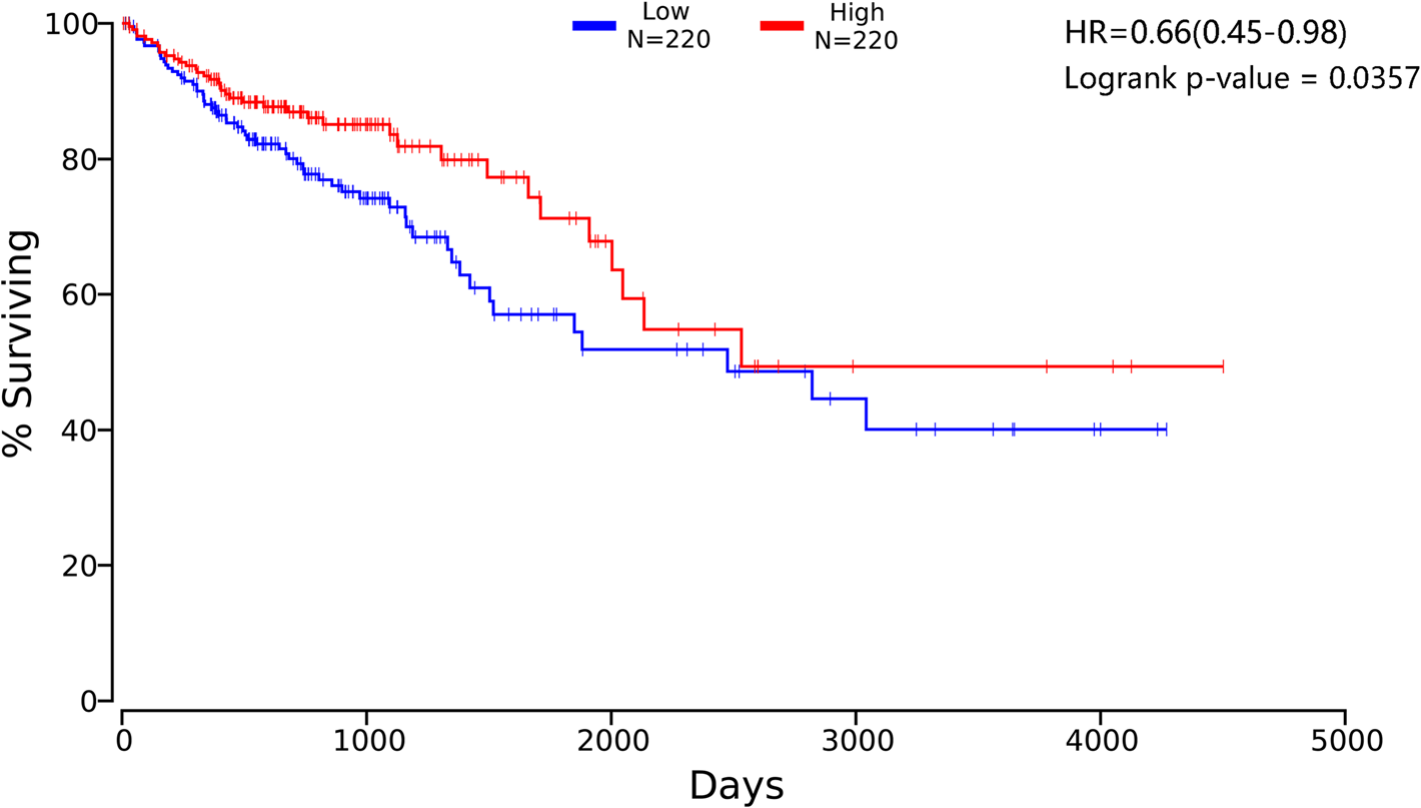
The association between COX-2 expression levels and overall survival of CRC

We evaluated the association between COX-2 gene expression and CRC prognosis using the OncoLnc website. Our data showed that high expression of COX-2 was associated with better OS for all CRC patients (HR, 0.66; 95% CI, 0.45–0.98; *p* = 0.0357, Fig. 2). We speculated that COX-2 may be a tumor suppressor gene.

**Fig. 3:**
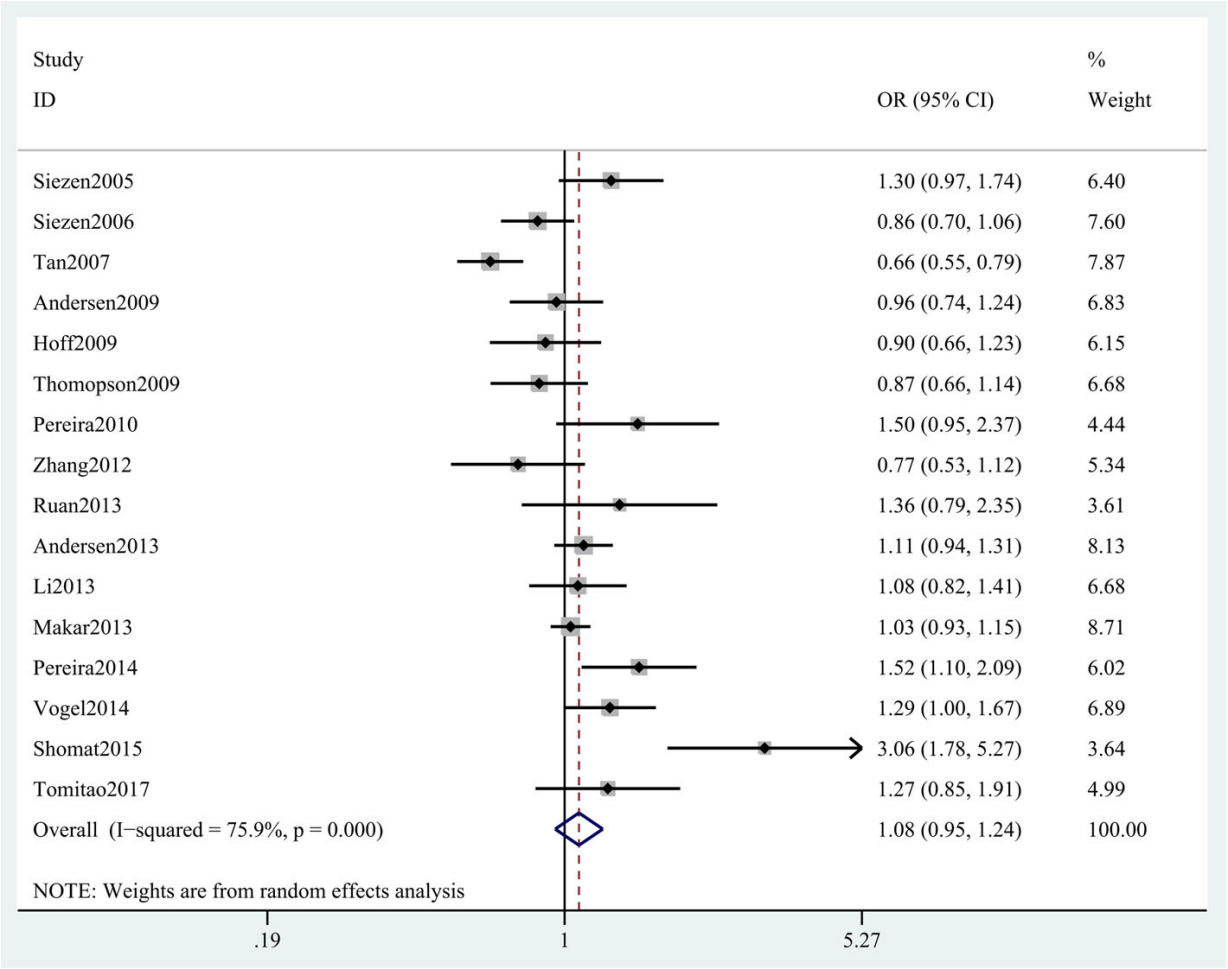
Forest plot shows odds ratio for the association between COX-2 rs689466 polymorphism and CRC risk (G vs. A)

**Fig. 4:**
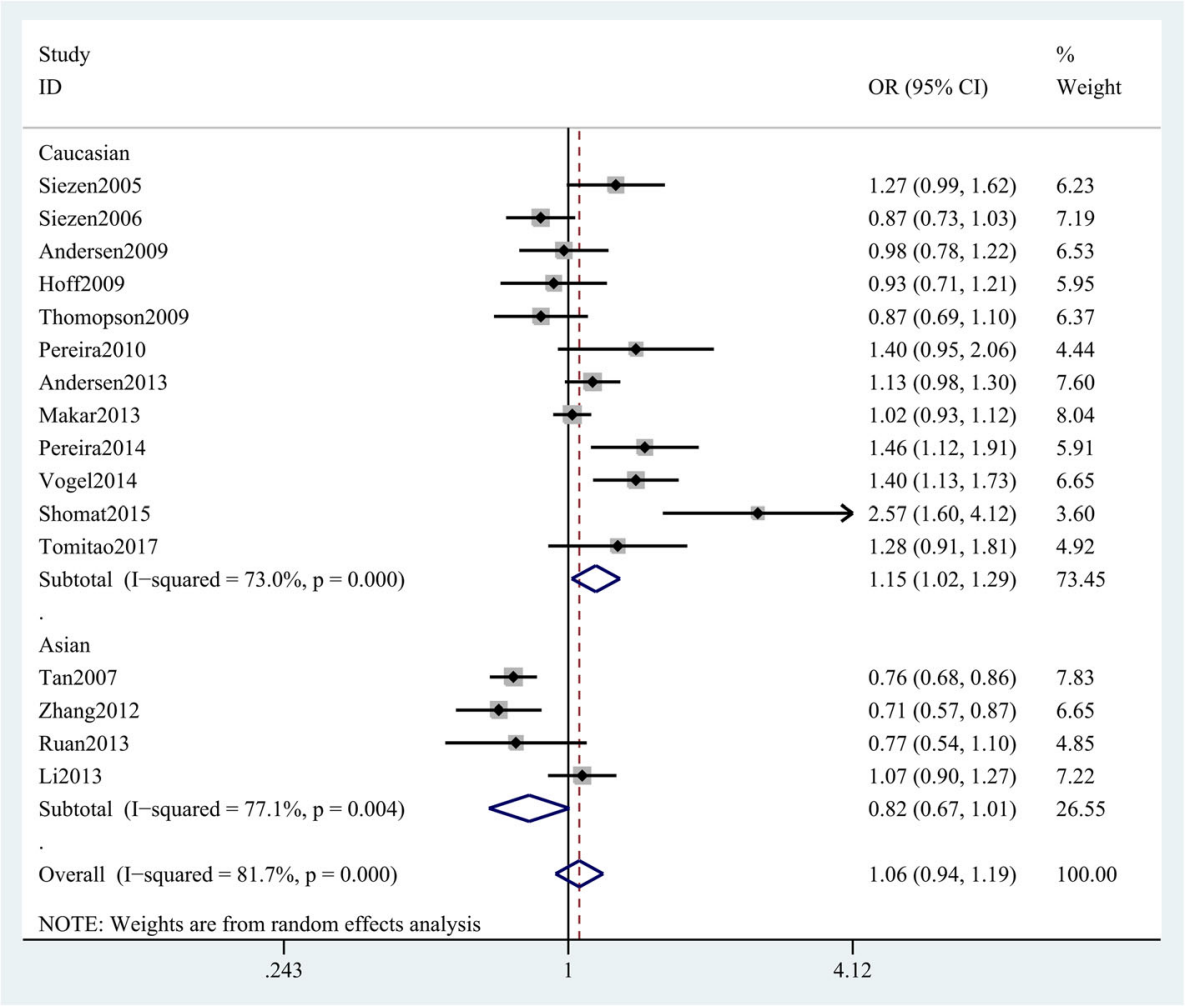
Stratification analyses of ethnicity between COX-2 rs689466 polymorphism and CRC risk (G vs. A)

We also conducted a meta-analysis between an important single nucleotide polymorphism (SNP) of COX-2 gene and CRC risk, and found that the COX-2 rs689466 polymorphism is not associated with CRC risk (G vs. A: OR = 1.06 (95% CI 0.94–1.19), *p* = 0.363, Fig. 3; GG + AG vs. AA 1.08 (0.95–1.24), *p* = 0.237; GG vs. AG + AA 1.06 (0.84–1.32), *p* = 0.627; GG vs. AA 1.10 (0.84–1.44), *p* = 0.478; GA vs. AA 1.07 (0.95–1.21), *p* = 0.453; Table 2). Nevertheless, an association between CRC risk and COX-2 rs689466 polymorphism was obtained in Caucasians (G vs. A OR = 1.15 (95% CI 1.02–1.29), *p* < 0.05, Fig. 4) but not in Asians. Stratified analysis by HWE and genotyping methods revealed no association either in HWE-positive studies (GG + AG vs. AA OR, 1.12 (95% CI 0.99–1.27), *p* = 0.068, Table 3) or HWE-negative studies. Stratified analysis of SOC showed that rs689466 polymorphism was associated with increased risk for hospital-based population. In addition, we found that rs689466 polymorphism was associated with the expression of COX-2 gene according to the result on GTEx portal data (Supplementary Figure 1).

**Table 2:** The association between COX-2 rs689466 polymorphism and colorectal cancer risk under different genetic models

Genetic models	OR (95% CI)	***p*** (OR)	Model	***I***^**2**^ (%)	***p*** (H)
Allele model (G vs. A)	1.06 (0.94, 1.19)	0.363	Random	81.7	< 0.001
Dominant model (GG + AG vs. AA)	1.08 (0.95, 1.24)	0.237	Random	75.9	< 0.001
Recessive model (GG vs. AG + AA)	1.06 (0.84, 1.32)	0.627	Random	66.8	< 0.001
Homozygous model (GG vs. AA)	1.10 (0.84, 1.44)	0.478	Random	73.5	< 0.001
Heterozygous model (AG vs. AA)	1.07 (0.95, 1.21)	0.257	Random	66.5	< 0.001

*OR* odds ratio, *CI* confidence interval, *p (H) p* for heterogeneity

**Table 3:** Meta-analysis of the association between COX-2 rs689466 polymorphism and colorectal cancer risk

Variables	No.	Allele model		Dominant model		Recessive model		Homozygous model		Heterozygous model	
		OR (95% CI)	*p*^het^	OR (95% CI)	*p*^het^	OR (95% CI)	*p*^het^	OR (95% CI)	*p*^het^	OR (95% CI)	*p*^het^
Ethnicity											
Caucasian	12	**1.15 (1.02, 1.29)**	< 0.001	**1.14 (1.00, 1.30)**	< 0.001	1.23 (0.99, 1.53)	0.138	1.28 (1.00, 1.65)	0.042	1.11 (0.98, 1.25)	0.005
Asian	4	0.82 (0.67, 1.01)	0.004	0.89 (0.65, 1.22)	0.006	0.76 (0.52, 1.11)	0.002	0.75 (0.46, 1.21)	0.001	0.94 (0.70, 1.26)	0.024
SOC											
PB	10	1.03 (0.91, 1.18)	< 0.001	1.01 (0.88, 1.16)	< 0.001	1.10 (0.86, 1.41)	0.001	1.10 (0.80, 1.52)	< 0.001	1.00 (0.88, 1.12)	0.002
HB	6	1.14 (0.84, 1.54)	< 0.001	1.32 (0.95, 1.83)	0.002	1.03 (0.60, 1.76)	0.001	1.17 (0.65, 2.10)	0.002	**1.33 (1.00, 1.78)**	0.022
HWE											
Positive	14	1.09 (0.96, 1.23)	< 0.001	1.12 (0.99, 1.27)	< 0.001	1.11 (0.85, 1.46)	< 0.001	1.17 (0.89, 1.55)	< 0.001	1.11 (0.99, 1.24)	0.008
Negative	2	0.90 (0.64, 1.26)	0.001	0.83 (0.52, 1.35)	0.003	0.90 (0.57, 1.40)	0.016	0.81 (0.40, 1.64)	0.001	0.84 (0.57, 1.25)	0.021
Genotyping											
Pyrosequencing	2	1.04 (0.72, 1.50)	0.014	1.04 (0.70, 1.56)	0.024	1.04 (0.58, 1.88)	0.135	1.07 (0.52, 2.19)	0.076	1.03 (0.72, 1.47)	0.058
PCR-RFLR	8	1.02 (0.83, 1.25)	< 0.001	1.11 (0.85, 1.44)	< 0.001	0.94 (0.67, 1.32)	< 0.001	0.99 (0.65, 1.51)	< 0.001	1.12 (0.88, 1.42)	< 0.001
Taqman	4	1.11 (0.87, 1.40)	0.005	1.10 (0.85, 1.42)	0.024	1.25 (0.80, 1.95)	0.281	1.28 (0.76, 2.16)	0.009	1.07 (0.86, 1.34)	0.116
Other methods	2	1.18 (0.86, 1.60)	0.008	1.12 (0.91, 1.37)	0.124	1.39 (0.62, 3.10)	0.018	1.44 (0.61, 3.38)	0.013	1.07 (0.96, 1.18)	0.333

*OR* odds ratio, *CI* confidence interval, *SOC* source of controls, *HB* hospital-based controls, *PB* population-based controls, *HWE* Hardy–Weinberg equilibrium, *PCR-RFLR* polymerase chain reaction-restriction fragment length polymorphism

### Sensitivity analysis and publication bias

**Fig. 5:**
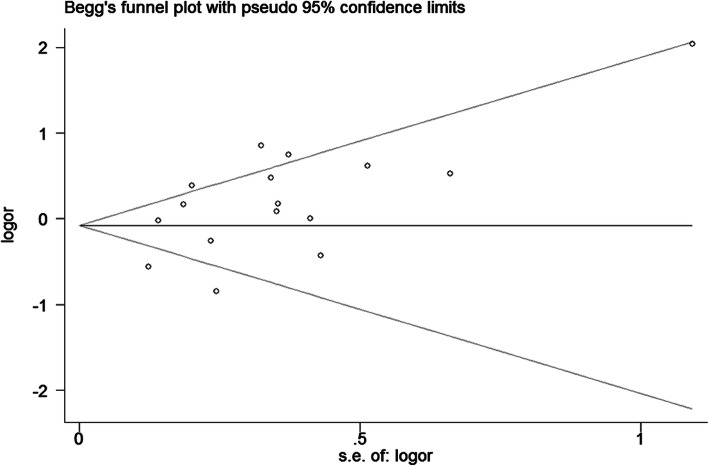
Begg’s tests for publication bias between COX-2 rs689466 polymorphism and CRC risk (GG + AG vs. AA)

Sensitivity analysis shows no single study largely influenced the pooled data, indicating that our results are statistically robust. Neither Begg’s funnel plot (GG + AG vs. AA, Fig. 5) nor Egger’s test finds any publication bias.

### Power analysis and FPRP analyses

The power analysis revealed that this study had a power of 89.8% to detect the effect of rs689466 polymorphism on CRC susceptibility among Caucasians, assuming an OR of 1.15. Table 4 presented the FPRP values at different *p* level. The FPRPs for positive associations were much larger, indicating some possible bias due to limited sample size. Larger-sized studies are needed to confirm these findings.

**Table 4:** False-positive report probability values for associations between COX-2 rs689466 polymorphism and colorectal cancer risk

Variables	OR (95% CI)	***p*** value	Power	Prior probability				
				0.25	0.1	0.01	0.001	0.0001
A vs. G								
Caucasian	1.15 (1.02, 1.29)	0.025	0.898	0.533	0.774	0.974	0.997	1.000
AA+AG vs. GG								
Caucasian	1.14 (1.00, 1.30)	0.046	0.800	0.561	0.793	0.977	0.998	1.000
AA vs. GG								
HB	1.33 (1.00, 1.78)	0.048	0.789	0.517	0.763	0.972	0.997	1.000

*OR* odds ratio, *CI* confidence interval, *HB* hospital-based controls

## Discussion

This meta-analysis showed no relationship between COX-2 rs689466 polymorphism and CRC risk in the whole populations. However, stratified analyses of ethnicity and SOC indicated that rs689466 polymorphism was associated with higher CRC risk among Caucasians and hospital-based populations.

CRC is the third leading cancer, but its occurrence and death rates vary largely among different areas in the world [30]. The lifetime risk of CRC development is ~ 5% in many regions [1]. About 45% of diagnosed CRC patients die, regardless of therapy [1]. The COX-2 mRNA levels are over-expressed in almost 80% of CRC patients [31]. COX-2 inhibitors are promising candidates for chemoprevention of CRC in clinic [32, 33]. The use of COX-2 inhibitor may help to improve the outcomes of stage III CRC patients [34]. Abovementioned data suggested that COX-2 may participate in the development of CRC. Rs689466 polymorphism is a pivotal SNP of COX-2 gene. The G allele rs689466 polymorphism was reported to transcriptionally activate COX-2 in colon cancer cells [29]. Thus, we assumed this SNP may be associated with the risk of CRC. Recently, a host of studies investigated the relationship between COX-2 rs689466 polymorphism and CRC risk [10–25]. A case-control study from the Netherlands observed no association between this SNP and CRC risk [16], which was consistent with the findings of some Caucasian studies. However, positive association was also obtained among other Caucasians [12, 18, 19]. Of the four studies from China, two studies found no association between COX-2 rs689466 polymorphism and CRC risk [11, 22], while the other two studies demonstrated a correlation between this polymorphism and lower CRC risk [10, 15]. To solve these inconsistencies, Wang et al. conducted a meta-analysis involving 5 studies (1854 cases and 2950 controls) and concluded COX-2 rs689466 polymorphism was not associated with CRC susceptibility [35]. Similarly, another meta-analysis also suggested that COX-2 rs689466 polymorphism was not associated with CRC risk in the overall population or in the stratified analyses of ethnicity, cancer location, SOC, or HWE [36]. We think the previous two meta-analyses have some limitations. Firstly, Wang et al. [35] omitted three studies meeting the inclusion criteria [10, 15, 16] and did not conduct stratified analyses of SOC or HWE. Secondly, Peng et al. omitted a study [36] and did not analyze the origin of heterogeneity. Therefore, their findings should be interpreted with caution. To date, several emerging studies have been reported since these meta-analyses. Consequently, it is necessary to conduct a comprehensive meta-analysis that included these new studies to determine whether the COX-2 rs689466 polymorphism was associated with CRC risk.

Herein, we included 16 studies with larger sample sizes (8998 cases and 11,917 controls) in this meta-analysis. Although our results suggested that COX-2 rs689466 polymorphism was not associated with a higher CRC risk in the overall population, subgroup analysis of ethnicity showed that COX-2 rs689466 polymorphism was associated with increased CRC risk in Caucasians, but not in Asians, suggesting different racial inheritance for Caucasians and Asians. The ethnic difference may be explained by the different allele frequency of this polymorphism. Asians have higher A allele frequency than Caucasians (European) (0.494 vs. 0.194). Another reason may be the differences among ethnic groups in sample sizes. In this meta-analysis, the sample sizes of Caucasians and Asians were different. Furthermore, varied living environments and diets may be important factors. In addition, clinical heterogeneity may also contribute to contradictory findings. As reported, G allele of rs689466 polymorphism could transcriptionally activate COX-2 [29]. We supposed this SNP may regulate COX-2 gene transcription and protein translation, thereby involving in the development of CRC. Additionally, we found that high expression of COX-2 was associated with better OS for CRC patients. To be frank, the development of CRC is attributed to multiple genes, genetic background, and environmental factors. Further studies that considered environmental and genetic factors were urgently needed.

This meta-analysis has several limitations. Firstly, subgroup analyses of age, sex, smoking, drinking status, or tumor size were not conducted due to data shortage. Secondly, estimates of confounding factors were unadjusted, which might affect the final results. Thirdly, possible gene-gene and gene-environment interactions were ignored because of data insufficiency. Fourthly, only Asians and Caucasians were included, and the findings may be inapplicable to other racial groups. Fifthly, we did not explore the association between rs689466 polymorphism and COX-2 protein. Lastly, only four Asian studies were included in this meta-analysis.

In conclusion, this meta-analysis confirms an association between COX-2 gene rs689466 polymorphism and increased CRC risk among Caucasians. Nevertheless, this finding should be validated by further studies in other ethnicities.

## Supplementary information

**Additional file 1: Figure S1.** The association between rs689466 polymorphism and the expression of COX-2 gene.

## Data Availability

The relevant data could be available when the corresponding author was contacted.

## Abbreviations

COX-2: Cyclooxygenase-2
CRC: Colorectal cancer
COXs: Cyclooxygenases
PGE2: Prostaglandin E2
OR: Odds ratio
CI: Confidence interval
OS: Overall survival
NOS: Newcastle-Ottawa
SOC: Scale source of control
HWE: Hardy–Weinberg equilibrium
