# Supplementary figures and images for: COX-2 gene rs689466 polymorphism is associated with increased risk of colorectal cancer among Caucasians: a meta-analysis
Source: World J Surg Oncol. 2020 Jul 30;18:192. doi: 10.1186/s12957-020-01957-x (PMC7391579; doi:10.1186/s12957-020-01957-x)

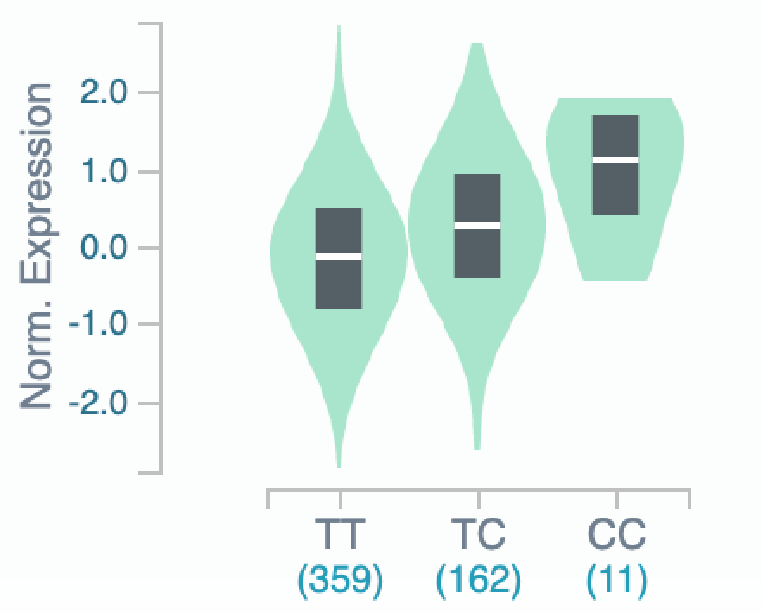

Supplement: Supplementary file 1 — Additional file 1: Figure S1. The association between rs689466 polymorphism and the expression of COX-2 gene. [file 12957_2020_1957_MOESM1_ESM.tif]
